# Supplementary material for: Prior Hypoxia Exposure Enhances Murine Microglial Inflammatory Gene Expression in vitro Without Concomitant H3K4me3 Enrichment
Source: Front Cell Neurosci. 2020 Oct 7;14:535549. doi: 10.3389/fncel.2020.535549 (PMC7575929; doi:10.3389/fncel.2020.535549)
Supplement: Supplementary file 1 [file Data_Sheet_1.docx]

Supplementary Material

**Supplementary Figure 1. Flow cytometry for cell viability.** (A) Cells were distinguished from debris using forward scatter (FSC) and side scatter (SSC). (B) Live cells were gated based on live/dead dye exclusion. (C) Single cells were identified by examining FSC height vs. FSC width. (D) N9 microglia were exposed overnight to 1-1.5% O_2_ (Hx) or to 21% O_2_ (room air; Nx). Cells were isolated for flow cytometry and stained with the live/dead dye eFluor780 to assess cell viability.

A.

B.

C.

**Supplementary Figure 2. Hypoxia primes microglial** **peptidoglycan-induced inflammatory gene expression.** A) Primary microglia were exposed to Nx or Hx overnight followed by 3 hrs LPS (100 ng/ml), and gene expression was examined using qRT-PCR. B) After 16 hrs of Hx cells were challenged with peptidoglycan (PGN; 30 μg/mL) for 3 hrs, and inflammatory gene expression was assessed by qRT-PCR. C) Wildtype mixed gene primary microglia were exposed to 16 hrs of Hx followed by 9 ug/mL recombinant HMGB1. . Results are expressed as the average fold change ± SEM of n = 3-8 independent experiments.†p < 0.01 vs. Nx vehicle; *p < 0.05 and **p < 0.01 vs. Nx LPS; Two-way RM-ANOVA.

**Supplementary Figure 3. STRING diagram for the 96 primed genes that had concomitant increases in H3K4me3 after Hx exposure.** Setting parameters were changed so that only high confidence connections are shown. All disconnected nodes were removed

**Supplementary Figure 4. Analysis of H3K4me3 peaks in β-glucan primed human monocytes compared to Hx primed microglia when peaks were called at 4-fold above background.** (A) Venn diagram showing overlap of H3K4me3 peaks in Hx primed vs. β-glucan primed cells. (B) Gene ontology for Biological Process demonstrated that both Hx primed microglia and β-glucan primed monocytes had H3K4me3 peaks at genes involved in cellular metabolism. Shared categories are in color.

**Supplementary Figure 5. MAGICTRICKS summary score for transcription factors regulating genes upregulated by Hx.** Analyses revealed Hif-1α as a transcription factor that binds genes enriched in the list of Hx upregulated genes.
